# Effects of Neuromuscular Electrical Stimulation on the Masticatory Muscles and Physiologic Sleep Variables in Adults with Cerebral Palsy: A Novel Therapeutic Approach

**DOI:** 10.1371/journal.pone.0128959

**Published:** 2015-08-06

**Authors:** Lilian Chrystiane Giannasi, Miriam Yumi Matsui, Sandra Regina Batista Freitas, Bruna F. Caldas, Eduardo Grossmann, José Benedito O. Amorim, Israel dos Reis dos Santos, Luis Vicente Franco Oliveira, Claudia Santos Oliveira, Monica Fernandes Gomes

**Affiliations:** 1 Bioscience, State of São Paulo University “Julio de Mesquita Filho”, UNESP/SJC, São José dos Campos, Brazil; 2 Rehabilitation Sciences Master and PhD Program, Nove de Julho University-UNINOVE, São Paulo, Brazil; 3 Anatomy Laboratory, Federal University of Rio Grande do Sul- UFRGS, Porto Alegre, RS, Brazil; University of Toronto, CANADA

## Abstract

Cerebral palsy (CP) is a term employed to define a group of non-progressive neuromotor disorders caused by damage to the immature or developing brain, with consequent limitations regarding movement and posture. CP may impair orapharygeal muscle tone, leading to a compromised chewing function and to sleep disorders (such as obstructive sleep apnea). Thirteen adults with CP underwent bilateral masseter and temporalis neuromuscular electrical stimulation (NMES) therapy. The effects on the masticatory muscles and sleep variables were evaluated using electromyography (EMG) and polysomnography (PSG), respectively, prior and after 2 months of NMES. EMG consisted of 3 tests in different positions: rest, mouth opening and maximum clenching effort (MCE). EMG values in the rest position were 100% higher than values recorded prior to therapy for all muscles analyzed (p < 0.05); mean mouth opening increased from 38.0 ± 8.0 to 44.0 ± 10.0 cm (p = 0.03). A significant difference in MCE was found only for the right masseter. PSG revealed an improved in the AHI from 7.2±7.0/h to 2.3±1.5/h (p < 0.05); total sleep time improved from 185 min to 250 min (p = 0.04) and minimun SaO2 improved from 83.6 ± 3.0 to 86.4 ± 4.0 (p = 0.04). NMES performed over a two-month period led to improvements in the electrical activity of the masticatory muscles at rest, mouth opening, isometric contraction and sleep variables, including the elimination of obstructive sleep apnea events in patients with CP.

**Trial Registration:**

ReBEC RBR994XFS http://www.ensaiosclinicos.gov.br

## Introduction

Cerebral palsy (CP) is a term employed to define a group of non-progressive neuromotor disorders caused by damage to the immature or developing brain, with consequent limitations regarding movement and posture [1]. Recent studies estimate a prevalence rate of two to five cases of out every 1000 live births, which represents a significant number of individuals with this condition [2].

Cognitive and motor function impairment, excessive muscle tone, fatigue, and an imbalance between agonist and antagonist muscles and compromised oral health are commonly associated with CP [3,4,5, 6,7,8]. Motor deficiencies in such cases are divided into primary (spasticity, movement quality, postural stability and the distribution of involvement) and secondary (strength, range of motion, endurance and movement limitations) [6]. Moreover, individuals with CP may also experience cognitive limitations, sensory deficits, joint deformities and chronic musculoskeletal pain [7,8]. Chewing, speaking and swallowing functions are often compromised. Dental caries and bruxism (clenching and/or grinding one’s teeth) are strongly associated with a reduction in oral health-related quality of life among individuals with CP [9]. As the impact on chewing function can directly or indirectly exert an influence on overall health, it is important to understand the behavior of oral movements in such individuals [9].

Surface electromyography (EMG) is one of the resources used to complement the clinical understanding of muscle function. With this method, electrical signals emitted from muscle cells are recorded to obtain information on muscle activation time, activity, strength and fatigue. Moreover, surface EMG is a reliable tool that allows measuring the effectiveness of therapies for muscle disorders, in both healthy and those with CP [10,11,12].

Neuromuscular electrical stimulation (NMES) is a promising therapy for strengthening muscles in patients with CP [13]. This safe, noninvasive method induces the action potential in motor nerves, causing the activation of motor units. NMES involves the administration of pulses of electrical current though electrodes (cathode and anode) placed over the muscle. The benefits of NMES include the preservation of muscle strength, a reduction in spasticity, improved flexibility and an increase in the range of motion of the affected limb, allowing voluntary efforts to become more effective; moreover, adverse effects are either nonexistent or negligible [14]. For individuals with CP, NMES has been recommended for the stimulation of muscles in the limbs and trunk and can lead to significant improvements in muscle strength and range of motion [13, 15,16] as well as the modulation of excessive muscle tone and enhanced coordination [17]. Considering the success of NMES regarding other muscle groups, it seems plausible that the use of this therapy on the masticatory muscles would be equally beneficial for the treatment of spasticity in this musculature. However, there are no records in the literature of studies addressing the effectiveness of NMES on the masticatory muscles in adult individuals with CP.

Neuromuscular impairment places patients with CP at risk for obstructive sleep apnea (OSA), which is characterized by the narrowing or obstruction of the upper airway during sleep. This progressive disease compromises quality of life and can lead to serious health problems and even death [18]. The pharyngeal muscles play a complex vital role in maintaining upper airway patency during sleep. The respiratory center stimulates pharyngeal muscle contraction to increase the pharyngeal tone just before diaphragmatic contraction. Otherwise the negative pressure in the pharynx would result in its collapse. This process is compromised in individuals with neuromuscular disorders. Poor neuromuscular tone plays a significant role in airway obstruction and increases with age. The worsening of airway obstruction/narrowing could be a result of the failure of hypotonic muscles to support the pharyngeal walls, leading to hypoventilation and hypoxemia during sleep, specially during REM sleep [19].

The prevalence rate of OSA is around 32% among normal adults of the city of Sao Paulo [20]. To the best of our knowledge, no studies have addressed the prevalence of OSA among adults with CP. Previous investigations have employed questionnaires to determine the prevalence of sleep disorders in children with CP and have demonstrated that children with both CP and OSA have compromised development and general health, reaching adulthood with a considerable motor and cognitive function limitations [21,22,23,24]. However, few studies have addressed the diagnosis and treatment of OSA in adults with CP [25,26,27] and investigations involving polysomnography (PSG) for the assessment of sleep patterns are very scarce [28,29]. Considering the magnitude of the consequences of OSA, it is important to assess sleep disorders in this population, as such disorders can lead to cardiovascular disease, metabolic problems, altered behavior and impairments with regard to daily functioning and learning.

The aim of the present study was evaluate the effects of NMES on the masticatory muscles and physiologic sleep variables in adults with CP using EMG and PSG, respectively. The hypothesis is the NMES will improve masticatory function and sleep variables.

## Material and Methods

The protocol for this trial and supporting TREND checklist are available as supporting information; see S1 TREND Checklist and S1 Protocol.

### 1. Subjects

**Fig 1:**
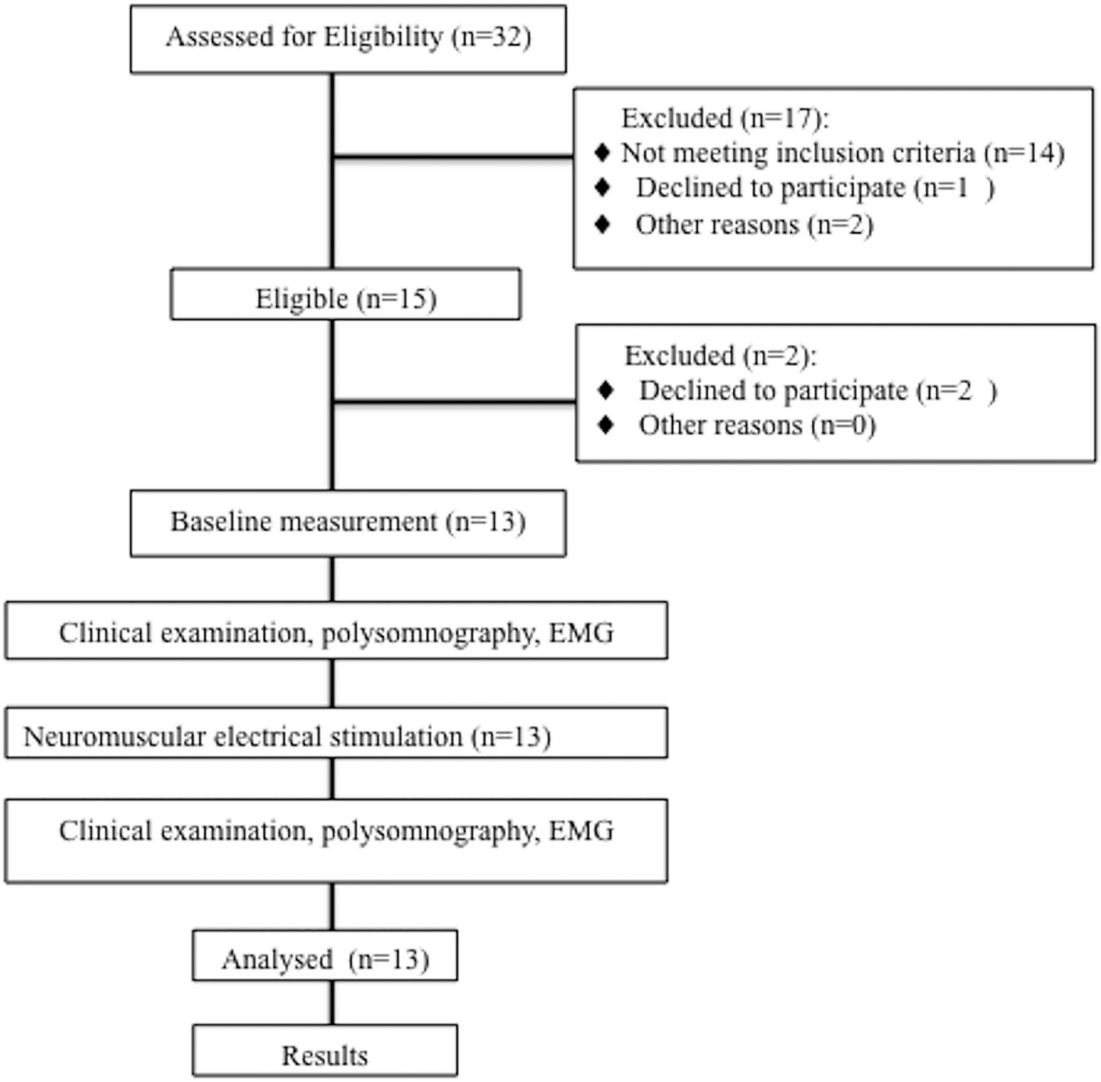
Flowchart of clinical study performed.

This work is a part of the study protocol that was approved and published prior to the onset of the present study. Initially, the protocol contained five arms, with 10 patients in each group. The objective was to evaluate the effects of the following therapies on the masticatory muscles and sleep variables: laser therapy, LED therapy, NMES, LED plus NMES and laser plus NMES. During the selection of subjects, which occurred in a period of nine months, 300 patients were evaluated at the Special Care Needs Clinic of the State of Sao Paulo University- (UNESP campus: Sao Jose dos Campos), which treats patients with CP, mental illness, autism, Down syndrome, cancer, multiple sclerosis and other degenerative diseases. Among those with CP, 32 were children and 23 were adults. At the end of the nine-months period of evaluation, only 13 of the adults with CP had partially preserved cognition and met the inclusion criteria to form a standardized sample with regard to the type of CP. Thus, the study was conducted with one of the initial five groups (Fig 1).

As only 13 subjects met the criteria, investigation was limited to the effects of NMES therapy on the masticatory muscles and sleep variables in adults with CP.

The inclusion criteria were spastic diparetic CP, partially preserved cognitive function (ability to respond to verbal commands, such as “open your mouth”, “close your mouth” and “clench your teeth”) and a statement of informed consent signed by the participant or legal guardian agreeing to voluntary participation in the study. The exclusion criteria were having undergone orthodontic or orthopedic treatment of the jaws or therapies to reduce spasticity (e.g., botulin toxin) in the six months prior to the study. Only thirteen individuals (8 males and 7 females) aged 25 to 48 years met the eligibility criteria. The sample was then classified using the Gross Motor Functional Classification Scale: two individuals on level I, two on level II, four on level III and seven on level IV. Due to the difficulty in composing a homogeneous sample, no control group was included.

This study received approval from the UNESP ethics committee (process number: 25000.058696/2010-74) and the Brazilian National Human Research Ethics Committee (CONEP number: 007/2011). The (Universal Trial Number) is U1111-1123-7969 and the study is registered with ReBEC (RBR-994XFS). All participants/guardians were properly informed regarding the objectives and procedures and signed a statement of informed consent prior to testing. The equipment used causes no harm to patients.

### 2. Electromyography

EMG signals were captured using an eight-channel module consisting of a conditioner with a band pass filter with cut-off frequencies at 20 to 500 Hz, an amplifier gain of 1000 times and a common mode rejection ratio > 120 dB. All data were acquired and processed using a 16-bit analog-to-digital converter with a sampling frequency 2 kHz. Active bipolar electrodes with a pre-amplification gain of 20 times were used.

#### 2.1. Procedure

EMG of the right and left masseter and temporalis muscles was performed prior to NMES as well as after one and two months of therapy. During the sessions, the volunteer was instructed to remain seated in a chair, feet apart, shoulders relaxed and hands resting on thighs in a well-illuminated, silent recording room in a comfortable position with eyes open and without head support. A short training period was conducted prior to beginning the tests to familiarize the volunteer with the activities. Explanations concerning the procedures and electrode placement were given and the volunteer trained mouth opening and maximum voluntary clenching effort of the masticatory muscles. Pre-gelled, self-adhesive, bipolar, silver-silver chloride electrodes were positioned over the right masseter (RM), left masseter (LM), right temporalis (RT) and left temporalis (LT) muscles, with an inter-electrode distance of 20 mm. The sites for the electrodes were shaved and cleaned with a cotton ball soaked in 70% alcohol to diminish impedance. The surface electrodes were placed bilaterally based on anatomical references and guided by the direction of muscle fibers at two points: the anterior temporalis muscle– 2 to 3 cm superoposterior to the lateral corner of the eye in the region of greatest evidence of muscle mass parallel to the muscle fibers, but with the sensor oriented perpendicularly; and the superficial portion of the masseter– 1 to 2 cm above the gonial angle of the mandible in the region of greatest evidence of muscle mass with the sensor parallel to the muscle fiber. A rectangular metallic electrode measuring 3 x 2 cm coated with Lectron II conductive gel (Pharmaceutical Innovations) to increase the conduction capacity and avoid interference from external noise was attached to the left wrist of the volunteer for reference. Readings were performed with the mandible in the resting position, as well as during mouth open and maximal clenching effort (MCE). In session 1 (test), three readings were performed in the resting position with a two-minute interval between readings. After three minutes, three readings were performed in mouth opening position with a five-minute interval between readings, using a goniometer to measure the mouth opening amplitude. After three minutes, three readings were performed during MCE, with a five-minute interval between readings, using a force transducer to measure the bite force. The signals were recorded for 10 seconds each under each condition. The same procedures were repeated after a one-week interval in session 2 (retest).

#### 2.2. Data processing

The EMG signals were processed using specific routines carried out in the Matlab program, version 7.1 (The MathWorks Inc., Natick, Massachusetts, USA). For MCE, a three-second period was selected through a visual inspection of the raw data. A moving window was used to select the EMG signals of the RT, RM, LT and LM muscles based on the greatest amplitude and regularity in the four muscles simultaneously. For the resting position, the entire 10-second period of the EMG signal was used in the analysis. The signals were analyzed using time domain (amplitude). The amplitude of the raw EMG signal was defined as the root mean square (RMS_raw_) calculated using a 200-ms moving window. The mean amplitude during the 3-s and 10-s trials recorded during MCE and in the resting position, respectively, was used for analysis. The amplitude of the EMG signal during MCE (RMS_raw-MCE_) was normalized by the mean amplitude of the three EMG signals recorded during 10 s in the resting position (RMS_raw-rest_) as follows: RMS_MCE_ = RMS_raw-MCE_/RMS_raw-rest_. This normalization procedure is an alternative for patients with neurological disorders (Soderberg & Knutson, 2000) [31]. The amplitude of the signal obtained in the resting position was expressed as the percentage of the mean RMS_raw-MCE_ recorded in the three readings, as follows: RMS_rest_ = (RMS_raw-rest_/RMS_raw-MCE_) x 100.

### 3. Neuromuscular electrical stimulation

The four-channel device was used. NMES was performed in compliance with standards 601 and 601-2-10 of the International Electrotechnical Commission, which lay down safety rules for electromedical devices and electrical stimulators, respectively. The patients underwent two weekly 20-minute sessions of NMES of the masseter and temporalis muscles for eight weeks (total of 16 sessions). The electrodes were placed over the motor point of the muscles. The following parameters were employed: pulse frequency of 50 Hz, pulse width of 250 μs and on/off ratio of five seconds of stimulation and ten seconds of rest for 20 minutes per session. As no data are available in the literature regarding the administration of NMEE to the masticatory muscles, these parameters were chosen based on electrical stimulation studies carried out on the lower limbs [32,33]. However, since the masticatory muscles are smaller, the application time was reduced, as patients in a previous test did not support a longer period. The parameters employed evoked a muscle response with minimal discomfort to the patients. NMES was expected to induce a visible muscle contraction without pain. The intensity of the current was chosen during the session based on the volunteer’s sensitivity. Maximum intensity reached ranged from 12 to 19 mA, which is very different from the intensity supported by the tibialis muscle (range: 28 to 44 mA) [30].

### 4. Polysomnography

Full-night PSG was performed prior to NMES and after two months of therapy using a digital system at the Sleep Laboratory of Nove de Julho University. All recording sensors were attached to the volunteer in a non-invasive manner using tape or elastic bands. The following physiological variables were monitored simultaneously and continuously: four channels for the electroencephalogram (EEG) (C3-A2, C4-A1, O1-A2, O2-A1), two channels for the electrooculogram (EOG) (EOG-Left-A2, EOG-Right-A1), two channels for the surface electromyogram (muscles of the submentonian region and tibialis anterior muscle), one channel for the electrocardiogram (derivation V1 modified), airflow detection via two channels through a thermocouple (one channel) and nasal pressure (one channel), respiratory effort of the thorax (one channel) and abdomen (one channel) via x-trace belts, snoring (one channel) and body position (one channel) via EMBLA sensors, arterial oxygen saturation (SaO_2_) and pulse rate via an EMBLA oximeter. All sleep stages were visually scored according to standardized criteria for investigating sleep. EEG arousals, sleep-related respiratory events and leg movements were scored in accordance with the criteria established by the American Academy of Sleep Medicine Manual for Scoring Sleep and Associated Events [34]. Each volunteer was instructed to remain as relaxed as possible and sleep naturally, as if at home. All signals were recorded continuously. The apnea index (AI), hypopnea index (HI), apnea/hypopnea index (AHI), sleep latency, REM sleep latency, sleep stages 1, 2 and 3 (N1, N2 and N3), REM, sleep efficiency (SE%), total sleep time (TST), arousal index, periodic limb movement (PLM), mean oxyhemoglobin saturation (mean SaO_2_) and minimum oxyhemoglobin saturation (minSaO_2_) were evaluated. Throughout the night, a technician monitored each volunteer with experience in PSG. This study was performed in a 14-month period.

### 5. Statistical methods

The sample size was calculated using STATA (version 12) based on a study entitled "Effect of spastic cerebral palsy on jaw-closing muscles during clenching"[30], assuming the effect found through electromyography of the masticatory muscles in patients with cerebral palsy. The intervention was considered to result in a minimum effect similar to the electromyographic activity of the masticatory muscles in children with mild motor impairment in comparison to those with severe motor impairment. Thus, based on the mean and standard deviation of 15.8 ± 5.2 and 25.2 ± 11.0 in children with severe and mild motor impairment, respectively, 11 individuals were deemed necessary for a bidirectional alpha and 80% test power, to which two individuals were added to compensate for possible dropouts. Thus the final sample was made up of 13 individuals.

The Kolmogorov-Smirnov test was employed to determine the distribution (normal and non-normal) of the EMG and PSG data. In the evaluations of the effects of NNES on sleep variables, HI, total sleep time, arousal index, N1, N3 and mean SaO2 demonstrated normal distribution and were evaluated using the paired t-test. The AI, AHI, sleep latency, PLM, index, N2, REM and minimum SaO2 demonstrated non-normal distribution and were compared using the Wilcoxon signed rank test. In the evaluation of the effect of NNES activity of the right and left masseter and temporalis muscles (RM, LM, RT, LT respectively), RT and LM at rest, LT, RM, LM and goniometry during mouth opening and RM, LM and dynamometry during MCE exhibited normal distribution and were submitted to one-way repeated-measures ANOVA and Tukey’s test. LT and RM at rest, RT during mouth opening and RT and LT during MCE demonstrated non-normal distribution and were evaluated using the repeated-measures Kruskal-Wallis test. The level of significance on all tests was 5% (α = 0.05). The data were organized and tabulated using the Minitab Release origin (14.2 Upgrade, USA) and p-values < 0.05 were considered indicative of statistical significance.

## Results

Thirteen volunteers were initially selected for the study. The final sample consisted of thirteen patients (7 men and 6 women), with a mean age of 28.0 ± 2.0 years, mean body mass index (BMI) of 25.0 ± 3.0 kg/m^2^ and mean neck circumference of 38.2 ± 1.8 cm.

### 1. Electromyography

After two months of NMES, RMS values in the rest position were 100% higher than values recorded prior to therapy for all muscles analyzed (p < 0.05) (Table 1), demonstrating improvements in electrical activity and a gain in muscle tone at rest. Caregivers reported improved chewing function and a visible reduction in drooling throughout NMES therapy. Mean mouth opening increased from 38.0 ± 8.0 to 44.0 ± 10.0 cm (p = 0.03), which likely led to greater comfort and efficiency while eating.

**Table 1:** EMG results prior to NMES as well as after one and two months of therapy. The results were obtained from a repeated measures test.

Muscles	Rest			Mouth Opening		MCE	
	Period	Mean/SD	p-value	Mean/SD	p-value	Mean/SD	p-value
RT(RMS)	Prior	17.4±9.5	p = 0.04*	59.6±38.3	p = 0.116	402.3±191.0	p = 0.926
	1month	21.1±11.0		94.9±86.1		483.0±248.0	
	2month	33.0±13.8		122.2±100.8		489.5±29.0	
RM(RMS)	Prior	13.4±5.9	p = 0.012*	59.0±30.5	p<0.001*	332.7±193.0	p = 0.02*
	1month	23.2±17.8		116.0±69.0		481.3±264.0	
	2month	29.0±6.6		114.7±69.0		480.1±259.0	
LT(RMS)	Prior	16.8±8.8	p = 0.03*	76.5±78.0	p = 0.57	372.1±186.0	p = 0.06
	1month	26.3±16.8		92.5±57.0		476.1±305.0	
	2month	30.2±11.1		83.4±55.0		419.6±255.0	
LM(RMS)	Prior	11.4±4.7	p = 0.001*	62.8±29.0	p<0.001*	417.7±298.0	p = 0.08
	1month	23.7±20.1		121.5±75.6		540.2±277.0	
	2month	32.9±17.5		116.4±48.6		461.7±276.0	
Goniometry (cm)	Prior			3.8±0.8	p = 0.03*		
	1month			4.4±1.0			
	2month			4.6±1.0			
MCE (Kgf)	Prior					70.5±14.5	p = 0.625
	1month					68.3±22.7	
	2month					65.9±22.4	

Note: RT = right temporalis; RM = right masseter; LT = left temporalis; LM = left masseter; MCE = maximum clenching effort

* p ≤ 0.05.

Moreover, statistically significant differences were found in the electrical activity of RM and LM in the mouth opening position (p < 0.001), with mean RMS values increasing from 59.0 ± 30.0 to 114.0 ± 69.0 and 63.0 ± 29.0 to 116.0 ± 48.0, respectively, between baseline and after two months of NMES. For MCE, a statistically significant difference in RMS values was only found in the RM (p = 0.02) after two months of NMES, whereas the improvement in the other muscles did not achieve statistical significance. All EMG results were obtained from a repeated measures test.

### 2. Polysomnography

Six of 13 volunteers exhibited OSA, prior the study (4 with mild OSA and 2 with moderate OSA). After two months of NMES, all six of these volunteers had a normal AHI (< 5.0/h) (Table 2).

**Table 2:** Effect of NNES therapy on 06 patients with OSA.

CP patients with OSA	AHI prior	AHI post
1	10.9	0.0
2	6.1	2.4
3	13.2	4.6
4	21.9	2.9
5	21.4	2.0
6	6.1	2.2
Mean	13.3	2.4
SD	7.1	1.5

Note: CP = cerebral palsy; AHI = apnea/hypopnea index; OSA = obstructive sleep apnea; SD = standard deviation.

For the entire sample (n = 13), mean AHI improved from 7.2±7.0/h to 2.3±1.5/h (p < 0.05). Total sleep time improved from 185 min to 250 min (p = 0.04). Statistically significant differences were also found for other sleep variables, such as minimum SaO2 (Table 3).

**Table 3:** PSG findings prior to NMES and after two months of therapy in 13 patients.

Sleep variables	Pre-treatment	Post-treatment	p-value
Apnea Index	6.3 ± 7.7	1.5 ± 1.4	p = 0.04*
Hypopnea Index	1.0± 1.3	0.8± 0.7	p = 0.74
AHI	7.2±7.0	2.3 ± 1.5	p = 0.02*
Total sleep time	185.4 ± 97.3	251.0 ± 79.5	p = 0.04*
Sleep Latency	38.7±42.0	36.0±55.0	p = 0.89
REM latency	102.2±61.6	123.4±60.0	p = 0.74
Arousal Index	13.1±11.7	12.0±8.3	p = 0.79
PLM Index	2.9±3.0	0.2±0.7	p = 0.001
N1	27.0±19.6	20.6±13.0	p = 0.38
N2	37.1±13.4	43.9±16.6	p = 0.27
N3	22.3±21.1	21.9±13.4	p = 0.95
REM	4.4±3.0	9.3±9.4	p = 0.07
Mean SaO_2_	96.4±1.8	95.8±1.2	p = 0.26
Minimum SaO_2_	83.6±3.0	86.4±4.0	p = 0.04*

Note: AHI = apnea/hypopnea index; REM = rapid eye movement; PLM = periodic limb movements; N1 = sleep stage 1; N2 = sleep stage 2; N3 = sleep stage 3; SaO_2_ = oxyhemoglobin saturation

* p ≤ 0.05.

## Discussion

This is the first study to apply NMES to the masticatory muscles in adults with CP and evaluate its effect on sleep patterns. In the current literature, this therapy has only been used to improve muscular tone in the upper and lower limbs, with important, positive outcomes reported [13,14,30]. The results of the present study lead to the hypothesis that the highly significant (100%) improvement in the resting position (p < 0.05) in all muscles studied (RM, RT, LM and LT) seems to be related to a reduction in drooling, which resulted from the significant increase in muscle tone of the jaw elevator muscles, thereby preventing the involuntary opening of the mouth. Moreover, through an as yet unknown pathway, the effect of NMES may be extrapolated to the suprahyoid and infrahyoid muscles, which would explain the improvement in the vertical movement of the mandible (p < 0.05) and may have contributed to a better swallowing pattern, as suggested in a recent article [35].

The initial RMS values for the RM, LM and LT were similar. Despite the increase in electrical activity in all muscles during MCE, a statistically significant difference was only found in the RM following NMES therapy. This balance in the masticatory muscles is an important result for individuals with CP and may be related to improvements with regard to eating solid and semi-solid foods as well as the visible reduction in drooling reported by the caregivers. However, while the 20-minute NMES protocol was sufficient to achieve masticatory muscle balance, it did not lead to a significant increase in bite force.

The effect of NMES on the masticatory muscles may also be reflected in the oropharyngeal muscles, with a positive effect on the AHI. Six volunteers exhibited mild to moderate OSA prior to therapy, with a significant reduction in the mean AHI after NMES therapy (p < 0.05), as all six volunteers had normal AHI values at the final evaluation. Although NMES was not administered directly to the muscles of the oropharynx, the protocol resulted in an improvement in OSA. As there are no references in the literature on the treatment of OSA through NMES, the hypothesis for this finding is an increase in motor units in the suprahyoid and infrahyoid muscles and upper airways through the peripheral electrical stimulation of the masticatory muscles.

With the exception of the ossicles of the middle ear, there are two movable bones in the head and neck region: the mandible and hyoid. These bones are situated at the C2 and C3 levels, respectively. Both are singular (unpaired with a contralateral bone) and are connected to each other through ligaments and muscles Thus, any change in the position of the mandible is reflected in the hyoid bone and vice-versa. The masticatory muscles (masseter, temporalis, medial and lateral pterygoid muscles) as well as the suprahyoid and infrahyoid muscles are attached to these bones. The masticatory muscles share innervation from the trigeminal nerve and have distinct functions (raising, protruding or performing lateral movements of the mandible) as well as initiating vertical mandibular movement. Another group is the suprahydoid muscles (mylohyoid, geniohyoid, stylohyoid and digastric), which work together and not as individual units. Innervation of the suprahyoid muscles comes from three different cranial branches (V, XII, VII and V, respectively). The digastric are main depressors of the mandible, whereas the other suprahyoid muscles raise the larynx and hyoid bone. The third group is the infrahyoid muscles (sternohyoid, omohyoid, sternothyroid and thyrohyoid), which are attached to the hyoid bone, sternum, clavicle and scapula and are innervated by the three first cervical nerves. The muscles lower the larynx, hyoid bone and floor of the mouth as well as stabilize the hyoid muscle.

To close the mouth, part of the masticatory muscles, with the exception of the inferior head of the lateral pterygoid, contract at the same time and relaxation of the suprahyoid muscle occurs, along with stabilization of the hyoid bone by the infrahyoid muscles. The inverse occurs when the mouth is opened, with the relaxation of the elevator muscles of the mandible and contraction of the suprahyoid muscles while the infrahyoid muscles stabilize the hyoid bone. Thus, chewing function depends on the state of contraction of muscle groups acting in an agonist and antagonist fashion. A fourth group of muscles is related to the pharynx. The stylopharyngeus is innervated by cranial nerve IX and its function is to raise and increase the diameter of the pharynx. The upper, middle and lower constrictors and palatopharyngeus share the same innervation as the pharyngeal plexus (cranial nerves IX and X) and elevate the pharynx and larynx to facilitate swallowing and breathing [36,37].

The peripheral use of NMES on the masticatory muscles in a continual, repetitive fashion likely led to a central response of the nuclei of each cranial pair located in the brain stem and upper portion of the cervical spine. Trigeminal alpha motor neurons can be either excited or inhibited by peripheral stimuli. These neurons send impulses to periphery muscles (the masticatory muscles in the present case as well as the hyoid, pharyngeal and cuticular muscles). In addition to these peripheral afferences, central responses of the superior motor neurons affect chewing function. Somatomotor centers are related to the pyramidal and extrapyramidal systems and the motor nucleus of the trigeminal nerve has synapses with motor axons. These two systems converge at the same point: the trigeminal motor neuron. It is possible that electrical stimulation may also act centrally, neuromodulating the altered muscle contractibility mechanism, recruiting a larger number of muscle fibers and normalizing the tone of the masticatory, hyoid and pharyngeal muscles [38,39]. Thus, NMES allows a selective repetitive afferent signal to the central nervous system and activates both the local muscles as well as the reflex mechanisms necessary for the reorganization of motor activity and movements that have been affected lesions in the superior motor neurons. Moreover, NMES leads to a generalized increase in electrical potential until reaching a balance between the excitatory and inhibitory pulses of agonist and antagonist muscles, stimulating deactivated motor neurons. This gives the patient the opportunity to experience the “normal movement” consciously and relearn this movement through repetition, thereby modulating muscle tone [40,41]. The improvement in sleep quality demonstrated by the follow-up PSG may have occurred due to these changes as well as the central release of the dimorphic and peripheral release of endorphins due to peripheral NMES, as occurs with transcutaneous electrical nerve stimulation. Despite the small sample size and short follow up, the enhancement in sleep quality was demonstrated by the improvement in OSA.

All muscles of the stomatognathic system are small and located very close to each other [42]. This intricate relationship may have resulted in the increased tone of the suprahyoid, infrahyoid and pharyngeal muscles. Thus, the improvement in OSA likely occurred through an indirect pathway that originated in the masseter muscles, reverberating through the suprahyoid and infrahyoid muscles and reaching muscles of the oropharynx, thereby preventing the occurrence of obstructive respiratory events during sleep. It is possible that the balance between agonist and antagonist muscles achieved with NMES allowed the mandible to remain closed during sleep and led to an improvement in oropharyngeal muscle tone, which, in turn, provided a stable tongue position, thereby avoiding the obstruction of the upper airways, as demonstrated by the improvement in minimum SaO2 and the reduction in drooling reposted by family members.

Another factor that may have contributed to the present findings was that the volunteers had a BMI within the ideal range, with no occurrence of overweight or obesity. This aspect likely facilitated the treatment of mild to moderate apnea in the present sample.

Few studies have investigated sleep patterns [28,29] and the treatment of OSA [26,27,43] in adults with CP. Using an oral appliance [26], continuous positive airway pressure (CPAP) [43] and medication [27], different authors have demonstrated how the treatment of OSA leads to an improvement in the quality of life in individuals with disabilities. A previous investigation involving the use of PSG on 103 patients reports that CP is not associated with short sleep [29]. The present findings are in disagreement with this statement, as all participants demonstrated a short sleep pattern at baseline, which was increased significantly following therapy with NMES (p = 0.04). This finding likely demonstrates a reduction in the hypotonicity of the muscles of the upper airways, with consequently greater comfort during sleep and an improvement in sleep quality, which has an effect on the quality of life of such patients, as adequate sleep exerts a positive impact on the metabolism, social aspects and learning skills.

Direct access to the muscles of the oropharynx is difficult and therefore few therapies can exert a direct effect on these muscles. For instance, speech therapy in normal individuals had demonstrated positive results, with reductions in the AHI from moderate to mild [44]. However, individuals with CP are often unable to perform the necessary exercises properly. The use of medication to improve muscle tone of the upper airways in patients with CP was employed decades ago and success was achieved with baclofen administered to the trachea and not directly to the muscles involved [27]. Yoshida et al. (1999) employed an oral appliance in five patients with disabilities (CP and Down syndrome) and found a reduction in OSA, but patient cooperation was difficult to achieve. In a recent clinical case of a patient with CP, an improvement in OSA was achieved using CPAP, but patient acceptance of the therapy was also difficult. While these treatment options are widely recommended in the literature, patient adaptation continues to be a drawback. The present results encourage the use of NMES to obtain an indirect effect on the upper airways, thereby offering a more comfortable option that is easy to administer for the treatment of OSA.

Another point worth mentioning in the present study was the significant increase in nadir oxygen saturation from 83.6 to 86.4% (p = 0.04) following therapy with NMES. Previous studies have also demonstrated improvements in oxygen saturation and OSA following speech therapy exercises involving the masseter muscles, which resulted in changes in the pharyngeal muscles that contributed to the reorganization of the facial pattern and greater muscle harmony, as orofacial muscles have functional interrelationships and interdependence [44]. The present findings demonstrate that stimulation of the massetter with NMES may have contributed to changes in the suprahyoid, infrahyoid and pharyngeal muscles, leading to the elimination of OSA in the sample of adult patients with CP. Intermittent hypoxia is one of the main symptoms of OSA [45], forcing the individual to become temporarily deprived of an adequate oxygen supply. The systematic repetition of this intermittent hypoxia over months or years is associated with arterial hypertension, heart disease and inflammation as well as deficient growth and development [45, 46]. The quality of cell activity is proportional to its oxygenation capacity and has a direct influence on the function of muscles, organs and the entire organism, with a direct impact on the development and adequate function of all organs. It is therefore essential that such events be avoided.

The present findings suggest that the administration of NMES on the masticatory muscles had a positive effect on neighboring muscles of the oropharynx. Thus, NMES is a promising treatment option for patients with CP and opens a new field of research, including the use of this therapy on individuals without CP. NMES could be employed to improve muscle tone and the function of upper airway muscles, thereby preventing the occurrence of sleep apnea. The authors are aware that the small sample size was a limitation of the present investigation, but studies involving adults with CP are rare in the literature and have a small number of participants [25,26,27]. It is important to consider the difficulty of such studies face in establishing an adequate sample size following the application of the eligibility criteria.

The present investigation is a pioneering study, as adult patients with CP were submitted to NMES administered to the masticatory muscles for the analysis of its effect on sleep patterns in this population of patients. The findings suggest that NMES of the temporalis and masseter muscles and, consequently, the trigeminal nerve, is an effective method for the treatment of hypotonicity of the pharyngeal muscles and OSA in individuals with CP. The long-term effects of NMES on muscle tone and OSA should be investigated in further studies involving a larger sample and longer follow-up period. This noninvasive, low-cost, simple, replicable method has thus far demonstrated no adverse effects. Most studies on the effects of NMES, sleep patterns and the treatment of OSA have involved children. However, adults with CP merit equal attention so that novel therapeutic options can be evaluated and implemented to improve the quality of life of this population of patients.

## Conclusion

NMES performed over a two-month period led to an increase in the electrical activity of the masticatory muscles at rest, as well as during mouth opening and isometric contraction. Moreover, NMES reduced the number of pathological respiratory events in all patients who initially demonstrated OSA, reaching an apnea-hypopnea index within the normal range. A significant improvement in oxygen saturation was also found. NMES may be a noninvasive option for the treatment of OSA in individuals with CP that do not accept oral appliance or CPAP terapies. Further studies with a larger sample size are needed to confirm these findings.

## Supporting Information

S1 TREND Checklist (PDF)

S1 Fig (TIFF)

S1 Protocol (PDF)
