# Supplementary figures and images for: Effects of Neuromuscular Electrical Stimulation on the Masticatory Muscles and Physiologic Sleep Variables in Adults with Cerebral Palsy: A Novel Therapeutic Approach
Source: PLoS One. 2015 Aug 6;10(8):e0128959. doi: 10.1371/journal.pone.0128959 (PMC4527754; doi:10.1371/journal.pone.0128959)

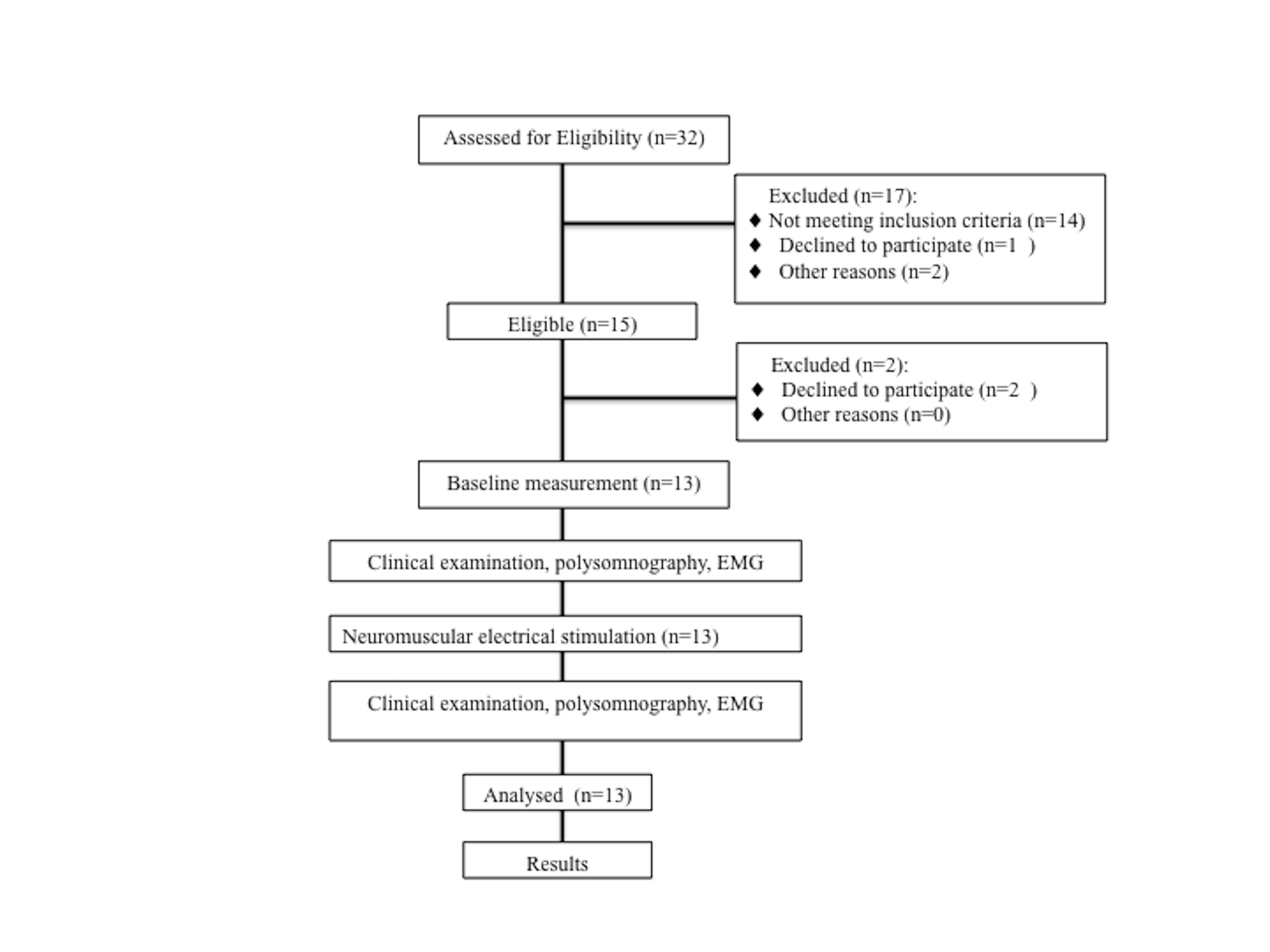

Supplement: S1 Fig — (TIFF) [file pone.0128959.s002.tiff]
